# Impact of contrast-induced acute kidney injury on patients with acute coronary syndrome: results of a prospective cohort

**DOI:** 10.3389/fcvm.2026.1777458

**Published:** 2026-04-08

**Authors:** Matheus S. Moitinho, Dulce Barbosa, Attilio Galhardo, Maximina Cunha, Adriano Caixeta, Beatriz Santana Prado, Eduesley Santana-Santos, Cassiane Dezoti Da Fonseca

**Affiliations:** 1 Heart Institute (InCor), São Paulo, Brazil; 2 Universidade Federal de Sao Paulo Escola Paulista de Enfermagem, São Paulo, Brazil; 3 Division of Cardiology, Institut Universitaire de Cardiologie et de Pneumologie de Quebec - Universite Laval, Québec City, Canada; 4 Division of Cardiology, Universidade Federal de Sao Paulo Escola Paulista de Medicina, São Paulo, Brazil; 5 Departamento de Enfermagem, Universidade Federal de Sergipe, São Cristóvão, Brazil

**Keywords:** acute coronary syndrome, acute kidney injury, cardiorenal syndrome, cardiorrenal syndrome, percutaneous coronary intervention

## Abstract

Contrast-induced acute kidney injury (CI-AKI) remains a relevant complication in patients with acute coronary syndrome (ACS) undergoing percutaneous coronary intervention (PCI), with important prognostic implications. This prospective cohort study evaluated factors associated with CI-AKI and its impact on short- to medium-term survival in patients with ACS treated with PCI. Patients were followed for 90 days between December 2019 and February 2021, with collection of sociodemographic, clinical, laboratory, and procedural data. CI-AKI incidence was analyzed using Poisson regression to estimate relative risks, and survival was assessed using a multivariable Cox proportional hazards model. 192 patients were analyzed, CI-AKI occurred in 33% of patients and was independently associated with advanced age, hypertension, chronic kidney disease, use of vasoactive drugs, and cardiorespiratory arrest. Mortality was 15% higher among patients who developed CI-AKI, and its occurrence was associated with a 4.7-fold increased hazard of death within 90 days, remaining an independent predictor after adjustment. These findings indicate that CI-AKI is a frequent and clinically relevant manifestation of cardiorenal interaction in ACS, strongly associated with increased medium-term mortality, underscoring the importance of early identification and preventive strategies in this high-risk population.

## Introduction

Cardiovascular diseases (CVD) remain a leading cause of morbidity and mortality worldwide, with coronary artery disease (CAD) representing one of its most prevalent manifestations. Common risk factors such as hypertension, diabetes mellitus, dyslipidemia, obesity, smoking, and sedentary lifestyle substantially increase the risk of CAD. In this context, percutaneous coronary intervention (PCI) has become a cornerstone therapy for coronary revascularization, particularly in patients with acute coronary syndrome (ACS), due to its rapid and minimally invasive nature (1). However, despite its therapeutic benefits, PCI carries a potential risk of Contrast-Induced Acute Kidney Injury (CI-AKI).

CI-AKI is a complex condition that has been extensively studied. Although the precise pathophysiological mechanisms remain incompletely understood, experimental studies suggest that CI-AKI involves contrast-induced hemodynamic changes, direct nephrotoxicity to tubular epithelial cells, osmotic nephrosis, and impaired oxygen distribution (2). Experimental and clinical studies have consistently demonstrated its association with adverse renal and cardiovascular outcomes. Established risk factors include advanced age, diabetes mellitus (DM), chronic kidney disease (CKD), and pre-existing cardiovascular disease) (2, 3). In patients with ACS, these risks are amplified by hemodynamic instability and the urgency of invasive procedures (3, 4).

Risk stratification tools have been developed to predict CI-AKI after PCI, with the Mehran risk score and its updated Mehran 2 model being among the most widely validated instruments in ACS populations (5). These scores incorporate clinical and hemodynamic variables to estimate renal risk and guide preventive strategies. Current preventive measures focus primarily on adequate periprocedural hydration, minimization of contrast volume, avoidance of nephrotoxic drugs, and, in selected patients, high-intensity statin therapy prior to PCI. Despite these strategies, CI-AKI remains a frequent complication in high-risk ACS patients (6).

From a clinical perspective, CI-AKI in the setting of ACS represents a manifestation of acute cardiorenal syndrome, in which acute cardiac dysfunction precipitates renal injury, leading to a bidirectional worsening of outcomes. Studies indicates that ACS patients are more susceptible to CI-AKI and experience worse outcomes (4). However, several key points warrant further investigation. There is a lack of comprehensive studies examining the risks of mortality and survival specifically in ACS patients with CI-AKI beyond in-hospital mortality (7, 8).

Moreover, many observational studies evaluating CI-AKI rely on logistic regression models, which estimate odds ratios that may overstate associations when outcomes are common (9–11). In longitudinal analyses, generalized linear models, such as Poisson regression, and time-to-event methods, including Cox proportional hazards models, provide more appropriate estimates of relative risk and hazard ratios, allowing for a more accurate assessment of prognostic impact (10).

Understanding the incidence, determinants, and prognostic implications of CI-AKI in patients with ACS undergoing PCI is essential to improve risk stratification and guide preventive strategies. Therefore, this study aimed to identify factors associated with the development of CI-AKI and to evaluate its impact on short- to medium-term survival in patients with ACS treated with PCI.

## Method

This study is a prospective longitudinal cohort analysis involving patients diagnosed with Acute Coronary Syndrome (ACS) who underwent urgent Percutaneous Coronary Intervention (PCI) at a tertiary referral academic hospital in São Paulo, Brazil. Conducted in alignment with the EQUATOR Network guidelines, the study adheres to the relevant reporting checklist for research reports.

Ethical approval was obtained from the institutional ethics and research committee under protocol number 3.763.447. The study population was enrolled from December 21, 2019, to February 28, 2021. Data collection involved patient interviews and the analysis of medical records. Patients were followed up for 90 days post-PCI to analyze mortality outcomes. During the COVID-19 pandemic period, operational constraints and data restructuring limited the availability of a complete screening log of all initially assessed patients. However, all consecutive eligible ACS patients undergoing PCI during the study period were included, and exclusions were restricted to cases with missing creatinine measurements, as reported in the Results section.

Inclusion criteria comprised individuals aged 18 years or older, diagnosed with ACS, who underwent PCI within 7 days of the acute event, remained hospitalized for at least 48 h after PCI in the tertiary center, and provided consent by signing the Informed Consent Form. Exclusion criteria included individuals with missing pre-exam serum creatinine values, or missing post-PCI creatinine values.

The glomerular filtration rate was estimated using the CKD-EPI equation (2021). CI-AKI was defined as an increase in serum creatinine (Cr) of ≥0.5 mg/dL or a relative increase of ≥25% from baseline between 48 and 72 h after the intervention, based on the KDIGO criteria. CKD was defined as a decrease in glomerular filtration rate below 60 mL/min/1.73m^2^ and/or the presence of abnormalities in renal structure lasting more than 3 months (12). All procedures were performed using low-osmolar non-ionic contrast media (Hemetix®).

The definitions for unstable angina, Non-ST Segment Elevation Myocardial Infarction (NSTEMI), and ST-Segment Elevation Myocardial Infarction (STEMI) were adopted from the Brazilian Society of Cardiology (1). The ischemic context was determined based on elevated necrosis biomarkers and electrocardiographic changes, in the presence of chest pain or its equivalents. Requiring diagnostic documentation by a cardiologist.

The Mehran score from 2021 (5) was employed as a variable to evaluate the risk of CI-AKI, considering ACS, renal function, Left Ventricular Ejection Fraction (LVEF), diabetes, serum hemoglobin, glucose levels, heart failure, and age >75 years.

The primary outcomes were the development of CI-AKI, in-hospital mortality, and time to mortality occurrence. Data were stored in a REDCap platform and transferred to a Microsoft® Excel® 2021 LTSC MSO spreadsheet for analysis.

Quantitative variables were tested for normality and homogeneity using the Shapiro–Wilk test and Levene’s test, respectively. Results were presented as mean or median (Md) with standard deviation (±) or Interquartile Range (IQR). Categorical variables were presented as absolute and relative frequencies (%).

The population was divided into two groups based on the occurrence of CI-AKI. For continuous variables, the Student’s *t*-test for independent samples (*t*) or the Mann–Whitney test (*U*) was used. For the analysis of the associations between categorical variables, the evaluation of the frequency distribution was used by the contingency table, thus, when the boxes obtained observations greater than five, Pearson’s chi-square test (*χ*^2^) was used. For cells with values lower than five, *χ*^2^ correction was used using the Yates method.

For the Cox model, the variables with *p* < 0.20 according to the univariate model were candidates for the multivariate model, in which the assumptions of multicollinearity and homoscedasticity were respected, with analysis of the Variance Inflation Factors (VIF) close to the value 1 and tolerance between the independent variables >0.8. Factors with high association or correlation were excluded from the final multivariate model.

A Poisson model with robust variance (9) and a log-link function was used to analyze factors associated with the incidence of CI-AKI, interpreting the estimators as RR.

Survival analysis was estimated using the Kaplan–Meier curve and compared using Log-rank, Gehan-Breslow, Tarone-Ware, and Peto-Peto tests. Prognostic factors for survival were identified using the Cox proportional hazards model for overall survival, with the estimators interpreted in terms of HR.

A 95% Confidence Interval (CI) was used, with a *p*-value <0.05 considered statistically significant. Effect size was evaluated using Cohen’s *d* (*d*), Cramer’s *V*, and biserial correlation of ranks (rb). Data analysis was performed using IBM® SPSS® Statistics version 21 and Jamovi 2.2.5®.

## Results

A total of 192 patients were included in this study. Nineteen patients were excluded due to missing pre- or post-procedural serum creatinine measurements and were therefore not included in the final analysis. The incidence of CI-AKI was 33% (*n* = 64) and was moderately related to advanced age (*p* < 0.012; *d*: −0.528), patients with Systemic Arterial Hypertension (SAH) (77.8%, *p* = 0.031; Cramer’s *V*: 0.165) and CKD (18.8%; *p* < 0.001; Cramer’s *V*: 0.246).

Although there were no statistically significant differences in the distributions of Acute ACS diagnoses related to CI-AKI (*p* = 0.481), it was observed that the troponin T value at the time of admission was 92 ng/mL (31–1,087 ng/mL) for those without CI-AKI and 329 ng/mL (41–431 ng/mL) for the CI-AKI group, indicating a statistically significant difference between the groups (*p* = 0.035; rb: 0.201) (Table 1).

**Table 1:** Sociodemographic characteristics of patients with acute coronary syndrome undergoing percutaneous coronary intervention, stratified by incidence of contrast-induced acute kidney injury, in São Paulo, Brazil, 2023.

Clinical-sociodemographic and PCI-related	total (*N* = 192)	CI-AKI		Statistical test
		NO	YES	
		(*N* = 128)	(*N* = 64)	
Male*_n_*_(%)_	137/192 (71.4%)	95/128 (74.2%)	42/64 (65.6%)	*χ*^2^**_(_**_1)_ = 1.54, *p* = 0.21^1^
Age_Mean (±)_	61.9 (10.6)	60.1 (10.8)	65.6 (9.2)	***t*_(190)_** **=** **−3.45, *p*** **<** **0.01**^2^
BMI_Md (IIQ)_	26.8 (24.4–29.8)	27 (24.6–29.8)	26.4 (23.8–29.7)	*U* = 3,826, *p* = 0.56^4^
Smoking*_n_*_(%)_				
Smoker/Ex Smoker	130/192 (67.7%)	91/128 (71.1%)	39/64 (60.9%)	*χ*^2^**_(_**_1)_ = 2.01, *p* = 0.16^1^
No Smoker	62/192 (32.3%)	37/128 (28.9%)	25/64 (39.1%)	
SAH*_n_*_(%)_	125/187 (66.8%)	76/124 (61.3%)	49/63 (77.8%)	***χ*^2^_(1)_** **=** **4.41, *p*** **=** **0.03**^1^
CKD*_n_*_(%)_	17/192 (8.9%)	5/128 (3.9%)	12/64 (18.8%)	***χ*^2^_(1)_** **=** **11.6, *p*** **<** **0.01**^1^
DM*_n_*_(%)_	58/192 (30.2%)	34/128 (26.6%)	24/64 (37.5%)	*χ*^2^**_(_**_1)_ = 2.42, *p* = 0.12^1^
Dyslipidemia*_n_*_(%)_	87/189 (46%)	56/127 (44.1%)	31/62 (50%)	*χ*^2^**_(_**_1)_ = 0.58, *p* = 0.44^1^
HF Preview*_n_*_(%)_	21/192 (10.9%)	11/128 (8.6%)	10/64 (15.6%)	*χ*^2^**_(_**_1)_ = 2.17, *p* = 0.14^1^
Diagnosing ACS*_n_*_(%)_				*χ*^2^**_(_**_2)_ = 1.45, *p* = 0.48^1^
Unstable angina	31/192 (16.1%)	23/128 (18.0%)	8/64 (12.5%)	
STEMI	98/192 (51.0%)	66/128 (51.6%)	32/64 (50.0%)	
NSTEMI	63/192 (32.8%)	39/128 (30.5%)	24/64 (37.5%)	
Troponin T Admission(ng/L)_Md (IIQ)_	136 (35–1,137)	92 (31–1,087)	329 (47–2,431)	***U*** **=** **2,875, *p*** **=** **0.03**^4^
KILLIP *n*(%)				
I–II	181/192 (94.3%)	122/128 (95.3%)	50/64 (92.2%)	*χ*^2^**_(_**_1)_ = 0.77, *p* = 0.39^1^
III–IV	11/192 (5.7%)	6/128 (4.7%)	9/64 (7.8%)	
Vasoactive drugs*_n_*_(%)_	21/191 (11.0%)	8/127 (6.3%)	13/64 (20.3%)	***χ*^2^_(1)_** **=** **8.54, *p*** **<** **0.01**^1^
Thrombolysis*_n_*_(%)_	25/191 (13.1%)	20/128 (15.6%)	5/64 (7.9%)	*χ*^2^**_(_**_1)_ = 2.19, *p* = 0.14^1^
Cardiorespiratory arrest*_n_*_(%)_	21/192 (10.9%)	6/128 (4.7%)	15/64 (23.4%)	***χ*^2^_(1)_** **=** **15.40, *p*** **<** **0.01**^1^
Hb Admission (mg/dL)_Md (IIQ)_	14.2 (12.6–15.8)	14.5 (13.0–15.9)	13.4 (11.9–14.9)	***U*** **=** **2,670, *p*** **<** **0.01**^4^
GFR Admission (mL/min)_Md (IIQ)_	81.0 (56.0–101)	65.2 (54.9–77.7)	60.6 (37.8–87.5)	*U* = 3,963, *p* = 0.72^4^
Cr Admission (mg/dL)_Md (IIQ)_	1.0 (0.82–1.2)	1.0 (0.9–1.2)	1.0 (0.8–1.5)	*U* = 3,982, *p* = 0.75^4^
Contrast Volume (mL)_Md (IIQ)_	150 (120–200)	150 (130–200)	150 (112–190)	*U* = 3,755, *p* = 0.75^4^
Number of stents_Md (IIQ)_	1 (1–2)	1 (1–2)	1 (1–2)	*U* = 3,505, *P* = 0.40^4^
ICP Duration_Md (IIQ)_	50.0 (35.0–65.8)	48.0 (34.9–65.0)	58.5 (35.4–70.0)	*U* = 3,370, *p* = 0.23^4^
Categories Mehran 2*_n_*_(%)_				
Low risk	13/192 (6.8%)	12/128 (9.38%)	1/64 (1.56%)	***χ*^2^_(3)_** **=** **16.52, *p*** **<** **0.01**^3^
Moderate risk	115/192 (59.9%)	84/128 (65.63%)	31/64 (48.44%)	
High risk	56/192 (29.2%)	30/128 (23.44%)	26/64 (40.63%)	
Very high risk	8/192 (4.2%)	2/128 (1.56%)	6/64 (9.38%)	

CI-AKI, contrast-induced acute kidney injury; SAH, systemic arterial hypertension; DRC, chronic kidney disease; DM, diabetes mellitus; BMI, body mass index; PCI, percutaneous coronary intervention; HF, heart failure; ACS, acute coronary syndrome; STEMI, ST-elevation myocardial infarction; NSTEMI, Non-ST-elevation myocardial infarction; Hb, serum hemoglobin; GFR, glomerular filtration rate; Cr, serum creatinine.

Bold values indicate statistical significance (*p* < 0.05).

^1^ Qui-quadrado de Pearson.

^2^ *t* de *student.*

^3^ Qui-quadrado com correção de Yates.

^4^ Mann–Whitney.

Additionally, the use of vasoactive drugs and Cardiorespiratory Arrest (CPA) were significantly associated with the outcome of CI-AKI (*p* < 0.01; Cramer’s *V*: 0.211; *p* < 0.01; Cramer’s *V*: 0.283) (Table 1).

The median and quartile values of Hb were lower for all measurement times (admission, post-24 h, and post-48 h) and were statistically discretely associated with the CI-AKI outcome (*p*: 0.005, rb: 0.258; *p*: 0.007, rb: 0.252; *p*: 0.013, rb: 0.238; respectively). There was no difference in admission Cr values among groups segregated by the presence of CI-AKI (*p*: 0.750) (Table 1).

Analysis of the Mehran 2 score revealed a small yet statistically significant difference in the incidence of CI-AKI (*p* = 0.001; rb = 0.278). Additionally, higher Mehran 2 scores were significantly associated with increased risk, falling into the high and very high-risk categories for CI-AKI (*p* < 0.001; Cramer’s *V* = 0.293), as shown in Table 1.

All clinical and sociodemographic variables were included in a univariate Poisson regression model to assess the incidence of CI-AKI. Factors associated with the incidence of CI-AKI, included age, SAH, CKD, vasoactive drugs, cardiorespiratory arrest and death within 90 days. For each additional year of age, the incidence of CI-AKI increased by 3% (RR: 1.034; 95% IC: 1.016–1.052). Patients with SAH had a CI-AKI rate 1.7 times higher than those without SAH (RR: 1.736; 95% IC: 1.042–2.891), those with CKD had rates 2.4 times higher (RR: 2.376; 95% IC: 1.621–3.481) and higher admission hemoglobin levels were associated with a reduced incidence of CI-AKI (RR: 0.897; 95% CI: 0.838–0.960). Individuals who required vasoactive drugs had 2 times higher rates of CI-AKI than those who did not (RR: 2.063; 95% IC: 1.122–3.794). Furthermore, individuals experiencing cardiopulmonary arrest and those who died within 90 days exhibited a 2.5-fold and 2.3-fold higher CI-AKI incidence, respectively (RR: 2.493; 95% IC: 1.398–4.445 and RR: 2.321; 95% IC: 1.262–4.267) (Table 2).

**Table 2:** Univariate poisson model with the factors associated with the incidence of CI-AKI in patients with acute coronary syndrome undergoing percutaneous coronary intervention, in São Paulo, Brazil, 2,023.

Variables	CI-AKI		*p*	RR	Confidence interval 95%	
	Without CI-AKI	With CI-AKI			Low limit	Upper limit
Age_Mean (SD)_	60.1 (10.8)	65.6 (9.2)	<0.001	1.034	1.016	1.052
SAH*_n_*_(%)_	76 (61.3)	49 (77.8)	0.034	1.736	1.042	2.891
CKD*_n_*_(%)_	5 (3.9)	12 (18.8)	<0.001	2.376	1.621	3.481
Hb Admission (mg/dL)_Md (IIQ)_	14.5 (13.0–15.9)	13.4 (11.9–14.9)	0.002	0.897	0.838	0.960
Vasoactive drugs*_n_*_(%)_	8 (6.3)	13 (20.3)	0.020	2.063	1.122	3.794
Cardiorespiratory arrest*_n_*_(%)_	6 (4.7)	15 (23.4)	0.002	2.493	1.398	4.445
Death*_n_*_(%)_	6 (4.7)	13 (20.3)	0.007	2.321	1.262	4.267

CI-AKI, contrast-induced acute kidney injury; RR, relative risk; SAH, systemic arterial hypertension; CKD, chronic kidney disease.

**Figure 1:**
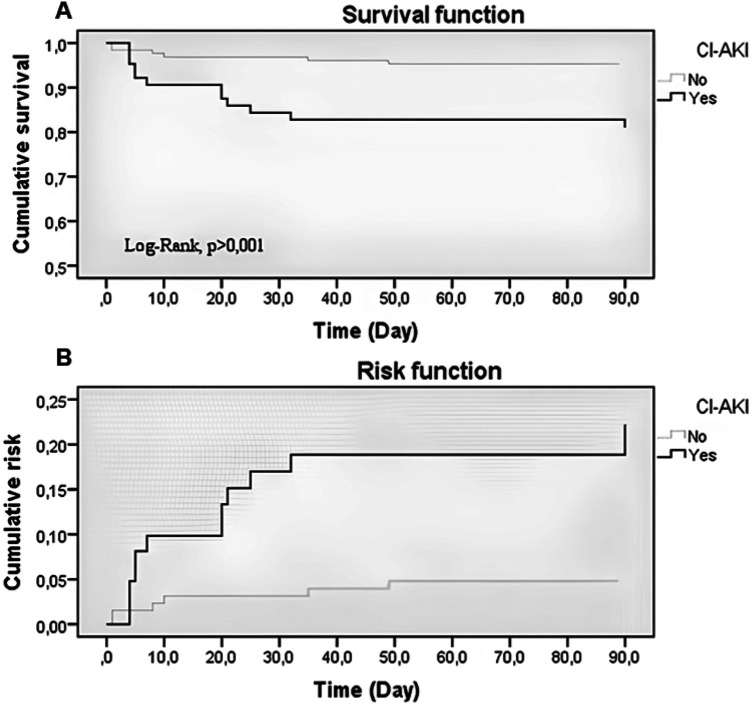
Kaplan–Meier analysis of cumulative survival and cumulative risk in patients with acute coronary syndrome undergoing percutaneous coronary intervention, stratified by contrast-induced acute kidney injury, São Paulo, Brazil, 2023. Kaplan–Meier estimates of cumulative 90-day survival **(A)** and incidence of death **(B)** based on the presence of Contrast-Induced Acute Kidney Injury.

At the 90-day follow-up, the mortality rate was 5% for patients without CI-AKI and 20% for those with CI-AKI. Analyzing the time until death, a significant difference is noted between the Kaplan–Meier survival curves. Patients without renal impairment showed significantly higher survival throughout the graph compared to those with CI-AKI (Log-rank, Gehan, Tarone-Ware, and Peto-Peto: *p* < 0.001 for all). Additionally, it is noteworthy that regardless of the presence of CI-AKI, the survival curve did not fall below 50% during the maximum observation period in either group (Figure 1).

In the Cox model with proportional risks, a slight aspect of non-proportionality of the data is observed immediately in the initial period of the horizontal axis of the Kaplan Meyer graph. However, after adjusting for covariates of the dime data to correct for non-proportionality, there is no significant loss noted between the inversions of the coefficients in the equation (Coefficient 1: *χ*^2^_(2)_: 0.00; *p* = 1.000; Coefficient 2: *χ*^2^_(2)_: 0.00; *p* = 1.000). Thus, it was decided to maintain the proportional hazards model. In this model, patients with CI-AKI have a significant approximately 4.7 times higher risk of death at the end of 90 day follow-up, compared to those without CI-AKI [HR: 4.67; 95% IC: 1.77–12.29, *p* = 0.002].

Analyzing survival rates over time, it is observed that patients without CI-AKI, there is a significantly higher survival rate (96.9%) at 30 days of follow-up compared to patients with CI-AKI (84.4%) [95% IC: 93.9%–99.93% vs. 75.9%–93.756%, respectively]. Similarly, noteworthy is the survival at 90 days, where patients without CI-AKI have a survival rate of 95.3%, while patients with CI-AKI have a survival rate of 79.7% [95% IC: 91.7%–99.04% vs. 70.4%–90.180%, respectively] (Table 3).

**Table 3:** Survival at 7, 30, 60 and 90 days, separated by the presence of CI-AKI, in patients with acute coronary syndrome undergoing percutaneous coronary intervention, in São Paulo, Brazil, 2023.

Levels	Time	Number at risk	Number of events	Survival	95% Confidence Interval	
					Lower	Upper
Without IRA-IC	7	126	2	98.4%	96.3%	100.0%
With IRA-IC	7	59	6	90.6%	83.8%	98.1%
Without IRA-IC	30	124	2	96.9%	93.9%	99.9%
With IRA-IC	30	54	4	84.4%	75.9%	93.8%
Without IRA-IC	60	122	2	95.3%	91.7%	99.0%
With IRA-IC	60	53	1	82.8%	74.1%	92.6%
Without IRA-IC	90	122	0	95.3%	91.7%	99.0%
With IRA-IC	90	52	2	79.7%	70.4%	90.2%

CI-AKI, contrast-induced acute kidney injury.

In the context of propositional hazards, a univariable and multivariable Cox regression model was executed to identify the factors associated with risks of mortality. The analysis revealed that several factors, including CI-AKI, KILLIP classification, use of vasoactive drugs, patient’s age, and Hb admission, were significantly associated with higher risk of death. Following adjustments for multicollinear factors, CKD and KILLIP classification were excluded from the final model. Notably CI-AKI [HR: 3.50; 95% IC: 1.01–12.14] and use of vasoactive drugs [HR: 4.17; 95% IC: 1.24–14.09] emerged as independent factors for the risk of death in this study population (Table 4). It is essential to emphasize that the model’s performance was statistically significant and demonstrated good concordance (Likelihood Ratio Test *χ*^2^_(7)_: 27.72; *p* < 0.001; Concordance: 0.814).

**Table 4:** Univariate and multivariate cox regression of time to death at 90 days in patients with acute coronary syndrome undergoing percutaneous coronary intervention, in São Paulo, Brazil, 2023.

	(Univariable)				(Multivariable)			
		95% CI				95% CI		
Variables	HR	Lower	Upper	*p*-value	HR	Lower	Upper	*p*-value
CI-AKI: No	Reference				Reference			
CI-AKI: Yes	**5**.**28**	**1**.**68**	**16**.**61**	***p*** **=** **0.004**	**3.50**	**1.01**	**12**.**14**	***p*** **=** **0.049**
CKD: No	Reference				—	—	—	—
CKD: Yes	3.03	0.96	9.52	*p* = 0.058	—	—	—	—
KILLIP: I-II	Reference				—	—	—	—
KILLIP: III-IV	5.78	1.83	**18.26**	***p*** **=** **0.003**	—	—	—	—
NSTEMI	Reference				Reference			
Unstable angina	0.64	0.07	6.15	*p* = 0.699	0.95	0.10	9.27	*p* = 0.964
STEMI	2.53	0.71	9.08	*p* = 0.154	2.53	0.71	9.08	*p* = 0.154
Vasoactive drugs: No	Reference	—	—	—	Reference	—	—	—
Vasoactive drugs: Yes	**8**.**33**	**3**.**01**	**23**.**06**	***p*** **<** **0.001**	**4.17**	**1.24**	**14**.**09**	***p*** **=** **0.021**
Age	**1**.**07**	**1**.**01**	**1**.**12**	***p*** **=** **0.015**	1.05	0.98	1.11	*p* = 0.148
Hb admission	**0**.**81**	**0**.**69**	**0**.**96**	***p*** **=** **0.016**	0.88	0.73	1.05	*p* = 0.154
LVEF Pre-intervention	0.98	0.94	1.01	*p* = 0.168	1.00	0.96	1.04	*p* = 0.971

HR, hazard ratio; CI, confidence interval; CI-AKI, contrast-induced acute kidney injury; DRC, chronic kidney disease; NSTEMI, non-ST-elevation myocardial infarction; STEMI, ST-elevation myocardial infarction; Hb, hemoglobin; LVEF, left ventricular ejection fraction.

Bold values indicate statistical significance (*p* < 0.05).

## Discussion

This study offers valuable information regarding the survival of patients with ACS undergoing PCI. Our findings support and strengthen the hypothesis that the presence of CI-AKI in these patients increases the risk of early mortality. Specifically, CI-AKI was identified as an independent predictor of higher mortality risk at 90 days. This aligns with Serdan et al. (13), who also found CI-AKI to be an independent predictor of long-term mortality (HR: 4.713; 95% CI: 1.53–14.51).

The adverse outcomes associated with AKI, regardless of its cause, are well-documented. Research has demonstrated that the incidence of AKI prolongs hospital stay (14), contributes to unfavorable outcomes (15, 16) and increases the chances of requiring dialysis and death (17, 18). The presence of AKI significantly raises mortality risk, and although this risk decreases over time (18), it remains elevated for up to 10 years even in patients who fully recover renal function (17).

The complexity and severity of ACS further increase susceptibility to AKI through various pathophysiological mechanisms (19). Previous studies have demonstrated the significant impact of acknowledging the interconnection between death, ACS, and AKI from all causes. A meta-analysis revealed that AKI mortality in ACS patients ranges from 0.8% to 18% (4) and AKI was independently associated with mortality, with a HR of 4.1 (95% CI: 3.3–5.0), indicating more than a fourfold increase in early mortality and more than a twofold increase in long-term mortality in ACS patients with AKI (4).

These findings underscore the critical need for specialized clinical attention in managing CI-AKI in ACS patients to improve clinical outcomes and long-term survival.

Our study also identified patient age, elevated KILLIP classification, the use of vasoactive drugs, and hemoglobin levels at admission as non-independent predictors of survival. Age, an independent risk factor for adverse outcomes, is included in key risk assessment tools (5, 20, 21). AKI showed associations with mortality, as evidenced in various studies (22, 23), including the study of Wi et al. (22), where Cr values >1.5 mg/dL were independent predictors for a higher risk of death (HR: 3.82; CI95%: 2.12–6.90), corroborating the findings of our present study.

Other predictors, such as KILLIP classification and the use of vasoactive drugs, are directly related to the clinical severity of patients, configuring a significant increase in the risk of death. The use of vasoactive drugs was also computed as an independent predictor of survival in our results, emphasizing the renal impact of the drugs or the clinic conditions necessary for them (24). Similarly, anemia, as demonstrated in previous studies, is a predictor of in-hospital mortality (6, 22). These results emphasize the need for a holistic approach in the assessment and management of these risk factors to improve clinical outcomes in patients with ACS.

Although STEMI is typically associated with greater infarct size and hemodynamic instability, ACS subtype was not independently associated with mortality after multivariable adjustment. This may reflect limited statistical power, but also suggests that markers of clinical severity, such as vasoactive drug use and KILLIP classification, may better capture prognostic risk than diagnostic category alone.

In our study, the incidence of CI-AKI was 33%, reflecting the high variability depending on lesion assessment criteria and the studied population. General PCI populations report CI-AKI incidences ranging from 3.3% to 14.5% (2). However, in ACS patients, the incidence ranges from 6.3% to 36.6% (4), highlighting the significant complexity of ACS and its influence on CI-AKI occurrence. Therefore, the reported incidence should be interpreted as representative of a high-acuity tertiary-care ACS population rather than an all-comer PCI cohort and may overestimate risk compared with broader registry-based populations, as our inclusion criteria required hospitalization beyond 48 h (to assess creatinine changes) and complete laboratory monitoring, potentially inflating the observed incidence.

Our study also examined the effects and associations of several variables with the incidence of CI-AKI Among these, advanced age had a notable impact, consistent with scientific literature that widely recognizes it as a risk factor for development of CI-AKI (3, 25). This is primarily due to the physiological decline in renal functional reserve and cellular regenerative capacity in older adults (26).

Associations such as SAH and CKD with the incidence of CI-AKI were also found and are exhaustively described in the scientific literature, emphasizing the importance of these comorbidities as risk factors for the development of AKI and CI-AKI (27, 28). Notably, a 2019 literature review summarized evidence that the presence of CKD moderates renal injury by exacerbating medullary hypoxia generated by tubular cytotoxicity, in addition to being responsible for the reduction of renal mass and contributing to endothelial dysfunction (27). A comprehensive meta-analysis involving over 169,455 patients identified hypertension as a factor associated with a higher risk of CI-AKI through meta-regression (28). hese findings underscore the importance of carefully considering comorbidities such as SAH and CKD when assessing CI-AKI risk in patients undergoing contrast procedures.

Furthermore, the presence of cardiopulmonary arrest and the need for vasoactive drugs were significantly associated with CI-AKI. Both variables are related to mechanisms and systemic repercussions of ischemia/reperfusion, hemodynamic instability, perfusional critical state, and systemic organ dysfunction, with a very high incidence of AKI, with rates reported between 32% and 47.8% according to some studies (24, 29, 30). It is known that kidneys are sensitive organs to hemodynamic instability, even if slight. Physiologically, they are organs that can trigger a decrease in GFR in the presence of mean arterial pressure below 80 mmHg due to renal hypoperfusion (31). More specifically, renal tubules operate in a borderline oxygenation regime, given the anatomical scarcity of descending vasa recta to the medullary region, making them very sensitive to small pressure variations (29, 31, 32). If hypoperfusion is severe and prolonged, or there is the presence of the aforementioned risk factors or exposure to contrast-induced nephrotoxicity, even moderate renal hypoperfusion can result in acute tubular necrosis (29, 32).

Studies have shown that renal blood flow initially increases after the administration of contrast agents but then undergoes abrupt vasoconstriction (32, 33). The reduction of renal blood flow, combined with marginal perfusion conditions, predisposes individuals to CI-AKI (29).

A notable finding in our study was the absence of a significant difference in the infused contrast volume between groups with and without CI-AKI This contradicts several studies emphasizing the critical role of iodinated contrast volume in CI-AKI (2, 6, 8, 32).

It is plausible that iodinated contrast has a smaller effect magnitude compared to clinical factors, which may explain the lack of this effect in our results. Our findings suggest that, in our specific population, factors such as patients’ baseline conditions and severity played more significant roles in CI-AKI incidence than contrast volume. This may reflect a predominance of patient-related risk factors and hemodynamic instability in our high-acuity ACS population, potentially generating a ceiling effect in which clinical severity outweighs procedural exposure. Additionally, contrast volume distribution was relatively homogeneous, limiting discriminatory power. Notably, the Mehran 2 score—validated in ACS patients—achieves excellent predictive performance without including contrast volume, further supporting the concept that in unstable cardiac populations, baseline clinical vulnerability may supersede procedural factors in determining renal risk. This underscores that, in the setting of ACS patients, contrast volume seems to have a lesser independent impact in the presence of clinical variables, although additional considerations are needed for a comprehensive understanding of this dynamic (5).

Also, different definitions of CI-AKI may substantially influence reported incidence and prognostic associations. The AKI criteria (≥0.3 mg/dL increase within 48 h) are more sensitive and could potentially yield higher incidence estimates in our cohort. However, the ≥0.5 mg/dL threshold used in this study may preferentially capture clinically meaningful renal injury, which may explain the strong association observed with mortality. Future studies comparing definitions within the same ACS cohort would help clarify the prognostic gradient associated with varying creatinine thresholds.

It is important to acknowledge the limitations of this study, including the potential overestimation of data due to the eligibility criteria requiring renal function evaluation in patients hospitalized for more than one day, potentially selecting more complex cases of ACS. The single-center nature of data collection and the relatively modest sample size also challenge the precision and generalizability of the results. Furthermore, we did not systematically collect data on non-fatal cardiovascular or renal adverse events during follow-up, which limits a more comprehensive assessment of the overall prognostic impact of CI-AKI beyond mortality. Also, the lack of extended follow-up complicates a comprehensive assessment of long-term implications, including mortality and clinical evolution. Future research should adopt a multicenter approach with larger and more diverse samples, allowing for extended follow-up and exploration of various outcomes. This would provide a more robust understanding of the multifaceted impact of CI-AKI in ACS patients, extending beyond strictly defined outcomes.

## Conclusion

The incidence of CI-AKI was a significant independent factor for the medium-term risk of death in patients with Acute Coronary Syndrome. Advanced age and clinical comorbidities played fundamental and independent roles as predictors for the occurrence of contrast-mediated renal injury. These associations underscore the multifactorial complexity of CI-AKI and its prognosis, emphasizing the necessity for a comprehensive approach in the clinical management of these patients.

## Data Availability

The datasets presented in this study can be found in online repositories. The names of the repository/repositories and accession number(s) can be found in the article/Supplementary Material.
